# Imaging of NKT Cell Recirculation and Tissue Migration during Antimicrobial Immunity

**DOI:** 10.3389/fimmu.2015.00356

**Published:** 2015-07-14

**Authors:** Terry L. Delovitch

**Affiliations:** ^1^Laboratory of Autoimmune Diabetes, Department of Microbiology and Immunology, Robarts Research Institute, Western University, London, ON, Canada

**Keywords:** NKT cells, anti-microbial immunity, immunoregulation, *in vivo* imaging, immunotherapy

This Opinion article outlines the relative paucity and emphasizes the need to enhance our knowledge of how subsets of natural killer T (NKT) cells mediate immune mechanisms of elimination of microbial pathogens at sites of inflammation or infection. To date, most studies of how NKT cell subsets migrate upon antigen stimulation have focused on NKT cell activation in the spleen, lymph nodes (LN) and liver (1). Thus, there currently exists an unmet need to determine the patterns of recirculation and tissue migration of NKT cell subsets and interacting antigen-presenting cells (APCs) that occur at relevant mucosal surfaces in several other organs, including the lung, intestine, and colon. This article proposes and highlights the benefit of *intravital cellular imaging in vivo* of type I and type II NKT cell subsets as an important methodology that may enable the visualization of NKT–APC cellular interactions at mucosal surfaces and enhance the application of this methodology to clinical therapy of antimicrobial immunity.

## T Cell Recirculation and Migration into Tissues

During an immune response, T cells and B cells traffic to and recirculate between blood and peripheral lymphoid tissues prior to activation by antigen (1). Chemokines attract T cells to various sites of interaction with antigen-presenting dendritic cells (DCs) in the spleen and LN. After further encounter with antigen, T cells divide and differentiate into effector T cells (Teff) that migrate to different sites of infection to combat and destroy microbial pathogens (2). Cytokines secreted by Teff also help to clear infectious pathogens from these sites. Interactions between T cells and DCs at various sites of inflammation in LN are crucial for promoting subsequent immunity to microbes (2). These observations underscore the importance of understanding how T cell recirculation, localization, and interaction *in vivo* in target tissues mediate effective immune responses that either trigger or prevent inflammation and antimicrobial immunity.

## Type I and Type II NKT Cell Subsets

Little is known about the various factors that mediate the recirculation, localization, and interactions of subsets of NKT cells *in vivo* in target tissues and lead to antimicrobial immunity. NKT cells display surface T-cell antigen receptors (TCR) expressed by both conventional T cells and NK cells, such as CD56/161 (humans) and NK1.1 (mice) (3–5). NKT cells recognize lipid antigens presented by CD1d MHC class I like molecules (2–15) on various APCs, including DCs, macrophages (Mϕ), B cells, thymocytes, adipocytes, and hepatocytes. While the CD1a, CD1b, CD1c, CD1e, and MR1 MHC class I like molecules are also expressed on APCs and can activate various T cell subsets, only analyses of CD1d-mediated responses of type I and type II NKT cell subsets will be presented here. The development of type I NKT cells occurs in the thymus and depends on the activity of several transcription factors including promyelocytic leukemia zinc finger (PLZF), T box transcription factor (T-bet), retinoic acid receptor-related orphan receptor-γt (ROR-γt), and GATA-binding protein 3 (GATA-3) (2, 5).

Type I NKT cells respond to α- and β-linked glycolipids. For example, stimulation of type I NKT cells by the α-galactosylceramide (αGalCer) glycolipid agonist induces the secretion of many cytokines that elicit both Th1 [interferon-γ (IFN-γ)] and Th2 [interleukin-4 (IL-4) and IL-13] responses (2, 7–17). Type I NKT cells are more prevalent than type II NKT cells in mice than in humans (18–20), and comprise about 50% of murine intra-hepatic lymphocytes (21–23). The type I NKT cell invariant TCR is encoded mainly by a germline Vα gene (Vα14/Jα18 in mice and Vα24/JαQ in humans), and more diverse non-germline Vβ chain genes (Vβ8.2/7/2 in mice and Vβ11 in humans) (1–20, 24–26). The semi-invariant TCR on type I NKT cells preferentially binds to CD1d via its α-chain (3, 6, 15, 25).

Type II NKT cells constitute a minor subset in mice, but are more predominant in humans (18, 27). Most type II NKT cells do not recognize α-linked glycolipids, but rather respond to sulphatide, a self-antigen that occurs naturally on cell membranes in the central nervous system (myelin sheath), pancreas, kidney, and liver. Sulphatide-reactive type II NKT cells may protect from autoimmune diseases by down-regulation of inflammatory responses elicited by type I NKT cells (28, 29). In contrast, non-sulphatide-reactive type II NKT cells may be pathogenic in other diseases, such as ulcerative colitis (UC) (30). Sulphatide-reactive type II NKT cells express oligoclonal TCRs and express a limited number of Vα and Vβ chains. The antigen specificity of type II NKT cells appears to be conferred by their surface TCR Vβ-chain (31).

## CD1d and NKT Cell-Mediated Antimicrobial Immunity

Antimicrobial defense may be mediated by extensive cross-regulation between CD1d, NKT cells, and microbes that function predominantly at mucosal surfaces (32–34). The display of microbes at mucosal surfaces, mainly during early postnatal development, controls NKT cell trafficking and function in the intestine, lung, and intestine. Microbial recognition at these sites determines the susceptibility to NKT cell-mediated inflammatory disorders. Conversely, CD1d expression controls the composition of the intestinal microbiota. Whereas microbiota reduce the number and activity of type I NKT cells at mucosal sites, an elevated number and function of type I NKT cells may be stimulated by microbiota in peripheral tissues (32). Thus, crosstalk between microbiota and type I NKT cells influences mucosal homeostasis and its dysregulation in a bidirectional manner in inflammatory disorders.

In human inflammatory bowel disease (IBD) and infectious hepatitis, type II NKT cells are causal to inflammation (10). In contrast, intestinal inflammation in oxazolone-induced colitis, a mouse model of human UC, is dependent on CD1d and type I NKT cells that express IL-17 and secrete IL-13 (10, 35). Thus, intestinal microbiota influence pathogenic responses in NKT cell-mediated intestinal inflammation. The outcome of these responses depends on the time of microbial exposure, NKT cell subset(s) involved, nature of microbial lipid antigens recognized, and type of APC that presents CD1d-restricted antigens to NKT cells. CD1d-restricted interactions of type I NKT cells with intestinal epithelial cells (IECs) promote IL-10 secretion and mucosal homeostasis, while CD1d-dependent interactions with bone marrow-derived APCs contribute to intestinal inflammation (36). Further experimentation may reveal whether these various responses result from the expression of different costimulatory molecules by IECs and professional APCs or whether cell-type-specific differences in CD1d trafficking and lipid acquisition contribute to this outcome. The central questions that need to be addressed are: (1) how do specific microbes control mucosal NKT cell abundance and function and determine health vs. disease, (2) what are the pathways of antigen-dependent and cytokine-dependent activation in NKT cells, and (3) do specific alterations in intestinal microbiota (e.g., in patients with IBD) (37) contribute to intestinal inflammation by the differential homing, proliferation, and activation of NKT cell subsets.

Like the intestine, the lung is a site of interaction between commensal microbiota and mucosal NKT cells. Insufficient microbial colonization during neonatal life leads to increased quantities and environmental sensitivity of type I NKT cells in lungs leading to susceptibility to asthma. This notion is supported by the result that exposure to antibiotics during early life but not late life enhances susceptibility to asthma in mice (38). In addition, elevated numbers of type I NKT cells are found in the lungs of germ-free mice. The latter finding requires the hypermethylation of the *Cxcl16* chemokine gene and increased expression of the CXCL16 chemokine protein, which binds to the CXCR6 cognate chemokine receptor found on NKT cells (39). These alterations are associated with increased airway resistance, eosinophil infiltration, and proinflammatory cytokine production during ovalbumin (OVA)-induced asthma in mice (39). Thus, the development, migration, and function of type I NKT cells at mucosal surfaces may be influenced by commensal microbiota (6).

## Tracking of T Cells *In vivo* by Intravital Cellular Imaging

Studies of NKT cell-mediated inflammation at different mucosal surfaces (e.g., intestine, lung, colon) illustrate that increased understanding of the mechanisms of differential recirculation, migration, proliferation, and activation of NKT cells during pathological responses requires the use of a technology that enables the visualization of these NKT cell events in real-time *in vivo*. The technique of two-photon (2P) microscopy coupled with *intravital* imaging enables one to track the location, movement, and interactions of cells (40–44). As such, 2P microscopy has improved our knowledge of T cell–DC and T cell–B cell interactions by recording how such cells function in resting tissue and undergo interaction, information exchange, and response to pathogens (40–43, 45). This methodology has also provided much new information about cellular pathways that arise during disease progression by illustrating the outcome of specific events in real-time (40–44). *Intravital imaging* and quantification of cell dynamics *in vivo* requires the use of fluorescently tagged proteins that are expressed transgenically in a cell-type-specific fashion to monitor the migration of single cells from blood vessels to tissues at a maximum tissue depth of 300–400 μm.

Initial studies on T cell–APC interactions during the establishment of peripheral tolerance were conducted with conventional CD4^+^ T cells and APCs in the LN and spleen, and showed that the time of contact between CD4^+^ T cells and APCs may vary from long-lived (days) to short-lived (a few hours) (40, 43). This difference in time of T cell–APC contact may influence the relative capacity of an agent administered *in vivo* to treat a given disease and induce (pre-disease) or restore (post-disease) immune tolerance. For example, CTLA-4 and PD-1 inhibitory receptors on Teff or regulatory T (Treg) cells can suppress immune responses by limiting the times of effective interactions of T cells with DCs (44, 46, 47). During chronic inflammation, cytokine delivery requires long-term T cell–APC contacts. However, only a relatively small number of cytokine molecules may be secreted at a low antigen concentration (43, 44, 46, 47). At a high concentration of antigen, the duration of T cell–APC contacts may be sufficiently long to elicit a chronic inflammatory response. Protection against inflammation is more likely to occur at a significantly lower antigen concentration (43). Further experimentation is required to analyze the effects of antigen concentration, time of cytokine production by CD4^+^ T cells in high vs. low antigen concentration tissue environments, and whether effector cytokines function locally at a particular site or are transported to other distal sites. Nonetheless, the results reported for the tracking and function of conventional CD4^+^ T cells *in vivo* have facilitated analyses of the migration and function of NKT cells *in vivo*.

## Imaging of NKT Cell Recirculation, Migration, and Activation

T cell receptor signal strength may determine the cytokine secretion profiles of T cells in a reciprocal manner. That is, the binding of TCRs of type I NKT cells to their antigen ligands can regulate the activity of TCRs on type II NKT cells. In turn, the binding of TCRs of type II NKT cells to their antigen ligands can regulate the activity of TCRs on type I NKT cells. Understanding the basis of how this cross-regulation of NKT cell activation occurs is crucial to develop better strategies to prevent microbial infection (2, 8–12, 48–52).

Such studies require a suitable animal model in which to track NKT cell recirculation and migration *in vivo*. For this reason, heterozygous mice were generated in which the green fluorescent protein (GFP) gene was knocked into a lineage-specific gene enabling certain leukocytes to be fluorescently labeled (53). In mice that express GFP integrated into the Cxcr6 chemokine receptor gene (Cxcr6gfp/+ mice), type I NKT cells traffic to, and become quite abundant in the liver (20–30% of lymphocytes). However, NKT cell migration within the liver is arrested following interaction with Kupffer cells. The latter interaction occurs within minutes following lipid antigen injection (54–58). In addition, both IL-12 and IL-18 proinflammatory cytokines induced following bacterial infection that suppresses type I NKT cell motility in liver sinusoids of Cxcr6gfp/+ mice via a CD1d-independent mechanism. This block in NKT cell movement is evident within 1 h after exposure to the cytokines and precedes NKT cell activation. Further antigen ligation stabilizes an immune synapse formed between NKT cells and interacting APCs. This synapse potentiates LFA-1/ICAM-1 interactions that enable activated type I NKT cells to remain in the liver. Thus, activated type I NKT cells recirculate less than activated conventional CD4^+^ T cells (59). Identification of the patterns and kinetics of recirculation of type I and type II mouse NKT cells as well as the patterns and kinetics of human type I and type II NKT cells await further study.

## Future Challenges

A future goal of studies of human NKT cells is to identify their functional roles in health and disease (1). Determination of how subsets of human NKT cells migrate and recirculate *in vivo* may advance our understanding of the biology and mechanisms of cellular interaction of different human NKT cells with APCs. Current investigations are being performed in two animal models. First, Cxcr6gfp/+ mice are being used to monitor human NKT cell trafficking, localization, and activation *in vivo* (56). Second, the kinetics and dynamics of human CD1d (hCD1d)-restricted NKT cell interactions are being analyzed in hCD1d knock-in mice that express hCD1d in place of mCD1d (59). Subpopulations of mouse type I NKT cells that are similar to human type I NKT cells in phenotype (mouse Vβ8^+^, human Vβ11 homolog^+^, CD4^low^), tissue distribution, and function (anti-tumor activity) are present in hCD1d knock-in mice. The latter mice serve to model how a lipid antigen induces the migration and function of hCD1d-restricted type I NKT cells and type II NKT cells *in vivo* (59–62). If type I and type II human NKT cells can be differentially activated or inhibited *in vivo*, this may facilitate the design of new immunotherapeutic protocols in the treatment and prevention of infectious diseases.

Additional imaging studies are required to delineate whether, in addition to NKT cells regulation at mucosal surfaces, commensal bacteria also regulate NKT cells at other sites, e.g., the skin where microbiota are in close contact with NKT cells and CD1a-restricted, lipid-reactive T cells (63–65). Future work may also establish potential species-specific and antigen-specific effects of microbiota on NKT cells and the roles of viruses and fungi in this process. Finally, it is of major clinical interest to develop therapeutic strategies that may induce changes in the function of type I NKT cells at mucosal surfaces that will promote and/or preserve mucosal homeostasis and antimicrobial immunity.

## Conflict of Interest Statement

The author declares that the research was conducted in the absence of any commercial or financial relationships that could be construed as a potential conflict of interest.
